# Perihelion Precessions of Inner Planets in Einstein's Theory and Predicted Values for the Cosmological Constant

**DOI:** 10.1155/2022/4808065

**Published:** 2022-10-28

**Authors:** Alvaro H. Salas S, Jairo E Castillo H, Lorenzo J. Martınez H

**Affiliations:** ^1^Universidad Nacional de Colombia, Bogotá, Colombia; ^2^FIZMAKO Researecg Group, Colombia; ^3^Universidad Distrital Francisco José de Caldas, Bogotá, Colombia; ^4^Department of Mathematics, Universidad Nacional de Colombia, Manizales, Colombia; ^5^Department of Mathematics and Statistics, Universidad de Caldas, Manizales, Caldas, Colombia

## Abstract

In this paper, we obtain the approximate value of 42.9815 arcsec/century for Mercury's perihelion precession by solving both numerically and analytically the nonlinear ordinary differential equation derived from the geodesic equation in Einstein's Theory of Relativity. We also compare our result with known results, and we illustrate graphically the way Mercury's perihelion moves. The results we obtained are applicable to any body that moves around the Sun. We give predictions about the value of the Cosmological Constant. Simple algebraic formulas allow to estimate perihelion shifts with high accuracy.

## 1. Introduction

For nearly a century, the consensus best theory has been Einstein's remarkably simple and elegant theory of general relativity [1]. This consensus is not without reason: practically all experiments and observations have lent increasing support to this theory, from classical weak-field observations such as the precession of Mercury's perihelion and the bending of starlight around the Sun, to the loss of orbital energy to gravitational waves in binary pulsar systems, observations remarkable both for their precision and for their origin in the strongest gravitational fields we have ever tested. Mercury is the inner most of the four terrestrial planets in the Solar System, moving with high velocity in the Sun's gravitational field. Only comets and asteroids approach the Sun closer at perihelion. This is why Mercury offers unique possibilities for testing general relativity and exploring the limits of alternative theories of gravitation with an interesting accuracy. Buoyed by his success, Le Verrier turned his sights to a planet whose orbit did not quite agree with Newtonian calculations: Mercury, the closest to the Sun. As is now famous, the perihelion of Mercury's orbit precessed at a slightly faster rate than was predicted by the Newtonian theory.

The precession of the orbit is not peculiar to Mercury, all the planetary orbits precess. In fact, Newton's theory predicts these effects, as being produced by the pull of the planets on one another. The question is whether Newton's predictions agree with the amount an orbit precesses; it is not enough to understand qualitatively what is the origin of an effect, such arguments must be backed by hard numbers to give them credence. The precession of the orbits of all the planets today can, in fact, be understood using the equations of the general theory of relativity.

As remarked in [1], the orbit of a planet around the sun can be found to a good approximation by considering the two-body interaction of that planet with the sun through an inverse square law central force. This leads to the familiar closed elliptical orbits of the planets studied in undergraduate physics and engineering classes. However, the presence of other planets causes the orbit to be not quite closed, the epsides slowly rotating or precessing in the plane of the orbit. In 1915, Albert Einstein published the final version of his theory of gravitation, called the “General Theory of Relativity” (GR), and calculated the additional displacement of the perihelion of Mercury resulting from it.

There are many works in which the perihelion precession of Mercury have been studied. In this work, we solve that equation both numerically and exactly and we present an algebraic formula for calculating perihelion precessions for all bodies that move around the Sun. The data used in this paper are presented in Table 1 [2]. The other data we will employ in this study are

(1) $$\begin{array}{c} {\text{Sun}}^{\prime }\text{ smass}:M=1.9889\times 10^{30}kg, \end{array}$$

(2) $$\begin{array}{c} \text{Gravitational constant}:G=6.67384\left(80\right)\times 10^{-11}.\frac{Nm^{2}}{Kg^{2}}, \end{array}$$

(3) $$\begin{array}{c} \text{Speed of the light in the vacuum}:c=299792458\frac{m}{s}. \end{array}$$

## 2. Equation of Motion

In the plane of the orbit, the radial distance *r*(*θ*) = 1/*u*(*θ*) of an object moving around the sun is given by [3, 4]

(4) $$\begin{array}{c} u^{\prime \prime }\left(\theta \right)=\frac{GM}{L^{2}}-u\left(\theta \right)+\frac{3GM}{c^{2}}u^{2}\left(\theta \right),\;u\left(0\right)=\frac{1}{P}\text{ and }u^{\prime }\left(0\right)=0, \end{array}$$

where *θ* is the polar angle and *L* is the angular momentum of the object (planet). Equation (4) is a Helmholtz equation. Its solution may be expressed in terms of elliptic functions [5] salast. If we take into account the cosmological constant Λ, the equation is modified by adding another term as follows [6]:

(5) $$\begin{array}{c} u^{\prime \prime }\left(\theta \right)=\frac{GM}{L^{2}}-\frac{c^{2}\Lambda }{3L^{2}u^{3}\left(\theta \right)}-u\left(\theta \right)+\frac{3GM}{c^{2}}u^{2}\left(\theta \right)=0,\;\;u\left(0\right)=\frac{1}{P}\text{ and }u^{\prime }\left(0\right)=0. \end{array}$$

**Figure 1:**
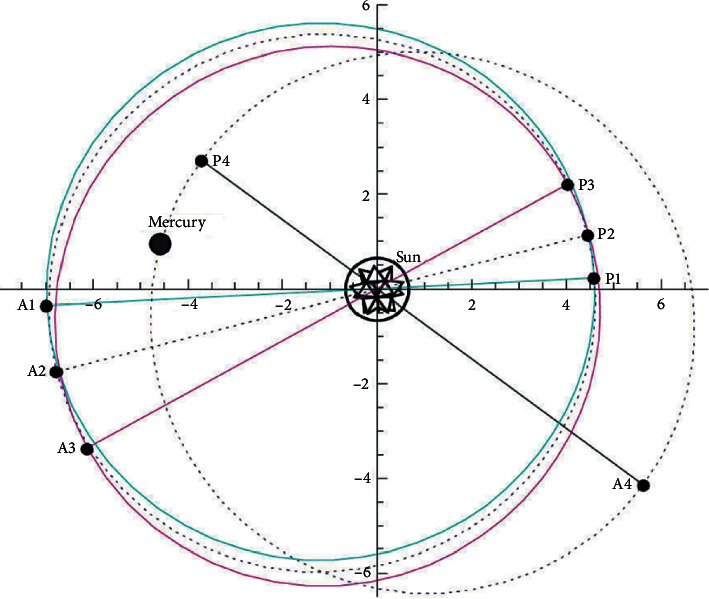
Advance of Mercury's perihelion.

Both equations (4) and (5) may be solved in closed form. However, equation (5) demands inverting some hyperelliptic Abelian integral and its solution is expressed in terms of a generalized Weierstrass function, which is a difficult task. We are interested in the value for the period of the solution to these equations. If we already found this value, the perihelion shift will then be simply Δ_*GTR*_=*T* − 2*π*.  Figure 1 illustrates the way Mercury's perihelion moves.

We will calculate exactly the value of *T*=*T*_Λ_ for the solution of (5). From these estimations, we will obtain the exact value of *T* for the period of the solution to (4) by letting Λ=0. Let us introduce the notations:

(6) $$\begin{array}{c} \alpha =\frac{GM}{L^{2}},\\ \beta =\frac{c^{2}\Lambda }{3L^{2}},\\ \gamma =\frac{3GM}{c^{2}},\\ \mu =GM. \end{array}$$

Then, the nonlinear differential equation to solve is

(7) $$\begin{array}{c} u^{\prime \prime }\left(\theta \right)-\alpha +\frac{\beta }{u{\left(\theta \right)}^{3}}+u\left(\theta \right)-\gamma u{\left(\theta \right)}^{2}=0,\;u\left(0\right)=\frac{1}{P}\text{ and }u^{\prime }\left(0\right)=0. \end{array}$$

Next, we multiply equation *u*^″^(*θ*) − *α*+*β*/*u*(*θ*)^3^+*u*(*θ*) − *γu*(*θ*)^2^=0 by *u*′(*θ*) and then we integrate it with respect to *θ* to obtain

(8) $$\begin{array}{c} \frac{1}{2}u^{\prime }{\left(\theta \right)}^{2}-\alpha u\left(\theta \right)-\frac{\beta }{2u{\left(\theta \right)}^{2}}-\frac{1}{3}\gamma u{\left(\theta \right)}^{3}+\frac{u{\left(\theta \right)}^{2}}{2}=C, \end{array}$$

where *C* is the constant of integration. Letting *θ*=0 and taking into account the conditions *u*(0)=1/*P* and *u*′(0)=0 gives

(9) $$\begin{array}{c} C=-\frac{\gamma }{3P^{3}}-\frac{\beta P^{2}}{2}+\frac{1}{2P^{2}}-\frac{\alpha }{P}. \end{array}$$

On the other hand, at the apelion position *θ*=*θ*_*A*_, we also have *u*(*θ*_*A*_)=1/*A* and *u*′(*θ*_*A*_)=0 so that

(10) $$\begin{array}{c} C=-\frac{\gamma }{3A^{3}}-\frac{\beta A^{2}}{2}+\frac{1}{2A^{2}}-\frac{\alpha }{A}. \end{array}$$

Using (9) and (10), we obtain

(11) $$\begin{array}{c} L^{2}=\frac{A^{2}P^{2}\left(6GM-Ac^{2}\Lambda P\left(A+P\right)\right)}{3AP\left(A+P\right)-2\gamma \left(A^{2}+AP+P^{2}\right)}. \end{array}$$

The angular momentum of the planet now depends on the contribution to it of the cosmological constant. Using the above relations, the hard nonlinear differential equation (5) takes the form:

(12) $$\begin{array}{c} d\theta =\pm \sqrt{\frac{3}{2\gamma }}\frac{u}{\sqrt{\left(1/P-u\right)\left(u-1/A\right)F\left(u\right)}}du\;,\\ F\left(u\right)≔-u^{3}+\frac{3AP-2\left(A+P\right)\gamma }{2AP\gamma }u^{2}-\frac{Ac^{2}P\left(A+P\right)\Lambda }{2L^{2}\gamma }u-\frac{Ac^{2}P\Lambda }{2L^{2}\gamma }. \end{array}$$

Thus,

(13) $$\begin{array}{c} T_{\Lambda }=2\sqrt{\frac{3}{2\gamma }}\int_{1/A}^{1/P}\frac{u\;du}{\sqrt{\left(1/P-u\right)\left(u-1/A\right)F\left(u\right)}}. \end{array}$$

Let

(14) $$\begin{array}{c} F\left(u\right)=\left(u^{2}-\varepsilon^{2}\right)\left(\rho -u\right), \end{array}$$

Since Λ is small, the number *ε* is small. Since 1/*A* ≤ *u* ≤ 1/*P*,

(15) $$\begin{array}{c} \frac{1}{\left(2A^{2}\right)}\left(\rho -u\right)<\left(u^{2}-\varepsilon^{2}\right)\left(\rho -u\right)<\frac{1}{P^{2}}\left(\rho -u\right). \end{array}$$

From (15), it follows that

(16) $$\begin{array}{c} \sqrt{\frac{3}{2\gamma }}P\frac{1}{\sqrt{\left(1/P-u\right)\left(u-1/A\right)\left(\rho -u\right)}}\\ <\sqrt{\frac{3}{2\gamma }}\frac{u}{\sqrt{\left(1/P-u\right)\left(u-1/A\right)F\left(u\right)}}\\ <\sqrt{2A^{2}\frac{3}{2\gamma }}\frac{1}{\sqrt{\left(1/P-u\right)\left(u-1/A\right)\left(\rho -u\right)}},\\ PG\left(\rho \right)<T_{\Lambda }<\sqrt{2}AG\left(\rho \right), \end{array}$$

where

(17) $$\begin{array}{c} G\left(k\right)=2\sqrt{\frac{3}{2\gamma }}\int_{1/A}^{1/P}\frac{u\;du}{\sqrt{\left(1/P-u\right)\left(u-1/A\right)\left(k-u\right)}}. \end{array}$$

This last integral has the exact value

(18) $$\begin{array}{c} G\left(k\right)=2\sqrt{\frac{3}{2\gamma P\left(\kappa P-1\right)}}\left(2\left(\kappa PK\left(\frac{A-P}{A-AP\kappa }\right)+\left(1-\kappa P\right)E\left(\frac{A-P}{A-AP\kappa }\right)\right)\right). \end{array}$$

From (15), we see that the quantity *T*_Λ_ may be approximated by

(19) $$\begin{array}{c} T_{\Lambda }\approx \frac{P+\sqrt{2}A}{2}G\left(\rho \right). \end{array}$$

Let *m*=*A* − *P*/*A* − *APκ*. We will consider two cases depending on the sign of the cosmological constant.

### 2.1. First Case: Λ > 0

(20) $$\begin{array}{c} F\left(u\right)=-u^{3}+\frac{3AP-2\left(A+P\right)\gamma }{2AP\gamma }u^{2}-\frac{Ac^{2}P\left(A+P\right)\Lambda }{2L^{2}\gamma }u-\frac{Ac^{2}P\Lambda }{2L^{2}\gamma }\\ <-u^{3}+\frac{3AP-2\left(A+P\right)\gamma }{2AP\gamma }u^{2}-\frac{Ac^{2}P\left(A+P\right)\Lambda }{2L^{2}\gamma }u\\ <-u^{3}+\frac{3AP-2\left(A+P\right)\gamma }{2AP\gamma }u^{2}-\frac{Ac^{2}P\left(A+P\right)\Lambda }{2L^{2}\gamma }u^{2}\\ =u^{-2}\left(\left[\frac{3AP-2\left(A+P\right)\gamma }{2AP\gamma }-\frac{Ac^{2}P\left(A+P\right)\Lambda }{2L^{2}\gamma }\right]-u\right)\\ =u^{-2}\left(d_{1}-u\right),\;d_{1}=\frac{3AP-2\left(A+P\right)\gamma }{2AP\gamma }-\frac{Ac^{2}P\left(A+P\right)\Lambda }{2L^{2}\gamma }>0. \end{array}$$

Then,

(21) $$\begin{array}{c} T_{\Lambda }>2\sqrt{\frac{3}{2\gamma }}\int_{1/A}^{1/P}\frac{du}{\sqrt{\left(1/P-u\right)\left(u-1/A\right)\left(d_{1}-u\right)}}≔\;\underline{\text{T}}\left(\Lambda \right). \end{array}$$

On the other hand, since *u*^−1^ ≤ *A* and *u*^−2^ ≤ *A*^2^,

(22) $$\begin{array}{c} F\left(u\right)=-u^{3}+\frac{3AP-2\left(A+P\right)\gamma }{2AP\gamma }u^{2}-\frac{Ac^{2}P\left(A+P\right)\Lambda }{2L^{2}\gamma }u-\frac{Ac^{2}P\Lambda }{2L^{2}\gamma }\\ =u^{2}\left(-u+\frac{3AP-2\left(A+P\right)\gamma }{2AP\gamma }-\frac{Ac^{2}P\left(A+P\right)\Lambda }{2L^{2}\gamma }u^{-1}-\frac{Ac^{2}P\Lambda }{2L^{2}\gamma }u^{-2}\right)\\ \geq u^{-2}\left(-u+\frac{3AP-2\left(A+P\right)\gamma }{2AP\gamma }-\frac{Ac^{2}P\left(A+P\right)\Lambda }{2L^{2}\gamma }A-\frac{Ac^{2}P\Lambda }{2L^{2}\gamma }A^{2}\right)\\ =u^{-2}\left(\left[\frac{3AP-2\left(A+P\right)\gamma }{2AP\gamma }-\frac{Ac^{2}P\left(A+P\right)\Lambda }{2L^{2}\gamma }A-\frac{Ac^{2}P\Lambda }{2L^{2}\gamma }A^{2}\right]-u\right)\\ =u^{-2}\left(d_{2}-u\right),\;d_{2}≔\frac{3AP-2\left(A+P\right)\gamma }{2AP\gamma }-\frac{Ac^{2}P\left(A+P\right)\Lambda }{2L^{2}\gamma }A-\frac{Ac^{2}P\Lambda }{2L^{2}\gamma }A^{2}>0. \end{array}$$

Then,

(23) $$\begin{array}{c} T_{\Lambda }<2\sqrt{\frac{3}{2\gamma }}\int_{1/A}^{1/P}\frac{du}{\sqrt{\left(1/P-u\right)\left(u-1/A\right)\left(d_{2}-u\right)}}≔\;\bar{\text{T}}\left(\Lambda \right). \end{array}$$

From (22) and (23), we have the following estimates:

(24) $$\begin{array}{c} \underline{T}\left(\Lambda \right)\leq T_{\Lambda }<\bar{T}\left(\Lambda \right). \end{array}$$

Then, we may approximate the value of *T*_Λ_ by means of the formula:

(25) $$\begin{array}{c} T_{\Lambda }=T_{\Lambda_{+}}=\frac{1}{2}\left(\underline{T}\left(\Lambda \right)+\bar{T}\left(\Lambda \right)\right). \end{array}$$

The integrals Ṯ(Λ) and T̄(Λ) may be evaluated with the aid of the exact formula:

(26) $$\begin{array}{c} 2\sqrt{\frac{3}{2\gamma }}\int_{1/A}^{1/P}\frac{du}{\sqrt{\left(1/P-u\right)\left(u-1/A\right)\left(d-u\right)}}=2\sqrt{\frac{6A}{\gamma \left(A\;d-1\right)}}K\left(\frac{A-P}{\left(A\;d-1\right)P}\right), \end{array}$$

where *K* is the elliptik function.

Let us check the accuracy of the obtained approximation in (25) for Mercury data *A*=69817332000 and *P*=46000870000 and assume that Λ=10^−51^. The numerical evaluation of the integral in (13) gives *T*_Λ_=6.283185782516926. On the other hand, making use of (26), we get

(27) $$\begin{array}{c} \left|\frac{1}{2}\left(\underline{T}\left(\Lambda \right)+\bar{T}\left(\Lambda \right)\right)-T_{\Lambda }\right|=2.653\times 10^{-8}. \end{array}$$

### 2.2. Second Case: Λ < 0

We have the following estimates for *F*(*u*):

(28) $$\begin{array}{c} F\left(u\right)=-u^{3}+\frac{3AP-2\left(A+P\right)\gamma }{2AP\gamma }u^{2}-\frac{Ac^{2}P\left(A+P\right)\Lambda }{2L^{2}\gamma }u-\frac{Ac^{2}P\Lambda }{2L^{2}\gamma }\\ >-u^{3}+\frac{3AP-2\left(A+P\right)\gamma }{2AP\gamma }u^{2}-\frac{Ac^{2}P\left(A+P\right)\Lambda }{2L^{2}\gamma }u\\ >-u^{3}+\frac{3AP-2\left(A+P\right)\gamma }{2AP\gamma }u^{2}-\frac{Ac^{2}P\left(A+P\right)\Lambda }{2L^{2}\gamma }u^{2}\\ =u^{-2}\left(\left[\frac{3AP-2\left(A+P\right)\gamma }{2AP\gamma }-\frac{Ac^{2}P\left(A+P\right)\Lambda }{2L^{2}\gamma }\right]-u\right)\\ =u^{-2}\left(d_{1}-u\right),\;d_{4}=\frac{3AP-2\left(A+P\right)\gamma }{2AP\gamma }-\frac{Ac^{2}P\left(A+P\right)\Lambda }{2L^{2}\gamma }>0. \end{array}$$

Then,

(29) $$\begin{array}{c} T_{\Lambda }<2\sqrt{\frac{3}{2\gamma }\int_{1/A}^{1/P}\frac{du}{\sqrt{\left(1/P-u\right)\left(u-1/A\right)\left(d_{4}-u\right)}}}≔T_{*}\left(\Lambda \right). \end{array}$$

On the other hand, since *u*^−1^ ≤ *A* and *u*^−2^ ≤ *A*^2^,

(30) $$\begin{array}{c} F\left(u\right)=-u^{3}+\frac{3AP-2\left(A+P\right)\gamma }{2AP\gamma }u^{2}-\frac{Ac^{2}P\left(A+P\right)\Lambda }{2L^{2}\gamma }u-\frac{Ac^{2}P\Lambda }{2L^{2}\gamma }\\ =u^{-2}\left(-u+\frac{3AP-2\left(A+P\right)\gamma }{2AP\gamma }-\frac{Ac^{2}P\left(A+P\right)\Lambda }{2L^{2}\gamma }u^{-1}-\frac{Ac^{2}P\Lambda }{2L^{2}\gamma }u^{-2}\right)\\ \leq u^{-2}\left(-u+\frac{3AP-2\left(A+P\right)\gamma }{2AP\gamma }-\frac{Ac^{2}P\left(A+P\right)\Lambda }{2L^{2}\gamma }A-\frac{Ac^{2}P\Lambda }{2L^{2}\gamma }A^{2}\right)\\ =u^{-2}\left(\left[\frac{3AP-2\left(A+P\right)\gamma }{2AP\gamma }-\frac{Ac^{2}P\left(A+P\right)\Lambda }{2L^{2}\gamma }A-\frac{Ac^{2}P\Lambda }{2L^{2}\gamma }A^{2}\right]-u\right)\\ =u^{-2}\left(d_{3}-u\right),\;d_{3}≔\frac{3AP-2\left(A+P\right)\gamma }{2AP\gamma }-\frac{Ac^{2}P\left(A+P\right)\Lambda }{2L^{2}\gamma }A-\frac{Ac^{2}P\Lambda }{2L^{2}\gamma }A^{2}>0. \end{array}$$

Then,

(31) $$\begin{array}{c} T\left(\Lambda \right)\geq 2\sqrt{\frac{3}{2\gamma }}\int_{1/A}^{1/P}\frac{du}{\sqrt{\left(1/P-u\right)\left(u-1/A\right)\left(d_{3}-u\right)}}≔T^{*}\left(\Lambda \right). \end{array}$$

The integrals *T*_*∗*_(Λ) and *T*^*∗*^(Λ) may be evaluated with the aid of the exact formula (26). Then, we may approximate the value of *T*_Λ_ by means of the formula:

(32) $$\begin{array}{c} T_{\Lambda }=T_{\Lambda_{-}}=\frac{1}{2}\left(T_{*}\left(\Lambda \right)+T^{*}\left(\Lambda \right)\right). \end{array}$$

Let us check the accuracy of the obtained approximation in (25) for Mercury data *A*=69817332000 and *P*=46000870000 and assuming that Λ=−10^−49^. The numerical evaluation of the integral in (13) gives *T*_Λ_=6.283185782516926. On the other hand, making use of (26), we get

(33) $$\begin{array}{c} \left|\frac{1}{2}\left(T_{*}\left(\Lambda \right)+T^{*}\left(\Lambda \right)\right)-T_{\Lambda }\right|=2.653\times 10^{-8}. \end{array}$$

## 3. Exact Value for Perihelion Shifts

In the limit when Λ_+_⟶0^+^, *d*_1_=*d*_2_=3*AP* − 2(*A*+*P*)*γ*/2*APγ* or when Λ_−_⟶0^−^, *d*_3_=*d*_4_=3*AP* − 2(*A*+*P*)*γ*/2*APγ* and then we obtain the exact value of *T* without considering the contribution of the cosmological constant:

(34) $$\begin{array}{c} T_{0}=\underset{\Lambda ⟶0^{+}}{\lim}T_{\Lambda }=2\sqrt{\frac{3}{2\gamma }}\int_{1/A}^{1/P}\frac{du}{\sqrt{\left(1/P-u\right)\left(u-1/A\right)\left(3AP-2\left(A+P\right)\gamma /2AP\gamma -u\right)}}. \end{array}$$

This last integral is expressed through the elliptik K function as follows:

(35) $$\begin{array}{c} T_{0}=\frac{4}{\sqrt{1-\varepsilon }}K\left(m\right),\;\text{where}\\ \varepsilon =\frac{2\gamma \left(A+2P\right)}{3AP}\text{ and }m=\frac{2\left(A-P\right)\gamma }{3AP-2\left(A+2P\right)\gamma }. \end{array}$$

The perihelion shift is easily obtained using the formula:

(36) $$\begin{array}{c} \Delta_{GTR}=\left(T_{0}-2\pi \right)\cdot \frac{23668612128}{\pi \cdot \text{Sidereal}}\;\text{arc sec}/\text{century}, \end{array}$$

where ‘Sidereal' stands for the sidereal period of the planet or object that moves around the sun. Using the approximations,

(37) $$\begin{array}{c} K\left(m\right)\approx \frac{\pi /2-5\pi m/32}{1-9m/16}\text{ and }\frac{1}{\sqrt{1-\varepsilon }}\approx \frac{1-\varepsilon /4}{1-3\varepsilon /4}. \end{array}$$

We get the following algebraic formula:

(38) $$\begin{array}{c} {\tilde{\Delta }}_{GTR}=\frac{4\left(8m+16\varepsilon -11m\varepsilon \right)}{\left(9m-16\right)\left(3\varepsilon -4\right)}\cdot \frac{23668612128}{\text{Sidereal}}\frac{\text{arc sec}}{\text{century}}. \end{array}$$

The above formula may also be written as

(40) $$\begin{array}{c} {\tilde{\Delta }}_{GTR}=-\frac{4\gamma \left(36P\left(Q+1\right)-\gamma \left(2Q+1\right)\left(13Q+35\right)\right)}{\left(24P-\left(\gamma 23Q+25\right)\right)\left(6P-7\left(\gamma +2\gamma Q\right)\right)}\cdot \frac{23668612128}{\text{Sidereal}}\frac{\text{arc sec}}{\text{century}}. \end{array}$$

Yet another more accurate formula in [7]:

(41) $$\begin{array}{c} {\hat{\Delta }}_{GTR}=\frac{71005836384\mu \left(c^{2}P\left(Q+1\right)+2\mu Q\left(Q+5\right)\right)}{c^{4}P^{2}\text{Sidereal}}\frac{\text{arc sec}}{\text{century}},\;\mu =GM,\;Q=\frac{P}{A}\;. \end{array}$$

The calculation of the perihelion shifts in arcsec/century for the planets is depicted in Table 2.

## 4. Estimated Theoretical Value for the Cosmological Constant

From (25), $T_{\Lambda }=T_{\Lambda_{+}}=1/2\left(\underline{T}\left(\Lambda \right)+\bar{T}\left(\Lambda \right)\right)$. The value of Λ is obtained by solving the transcendental equation $1/2\left(\underline{T}\left(\Lambda \right)+\bar{T}\left(\Lambda \right)\right)=T_{0}$, where *T*_0_ is given by (35). The values for Ṯ(Λ) and T̄(Λ) are obtained from (26) and (??). The results of calculations are shown in Table 3.

The geometric mean of the values in Table 3 equals Λ_+_=1.6511 × 10^−52^. So, our prediction for a positive cosmological constant is

(42) $$\begin{array}{c} \Lambda =1.6511\times 10^{-52}. \end{array}$$

## 5. Analysis and Discussion

Perihelion precessions of Mercury and other bodies have been the subject of experimental study from AD 1765 up to the present. In 1882, Simon Newcomb obtained the value 43 seconds per century for the discrepancy for Mercury [8]. According to Pireaux et al. [9], the observed advance of the perihelion of Mercury that is unexplained by Newtonian planetary perturbations or solar oblateness is

(43) $$\begin{array}{c} \Delta_{\text{obs}}=42.980\pm 0,002\text{ arc}-\text{second per century.} \end{array}$$

For the case of the planet Mercury and without taking into account the cosmological constant, the theoretical results coincide with the experimental results. Some authors claim that the correction of the perihelion precession of Mercury induced by the cosmological constant is ruled out at many levels of *σ* [10]. In this work, we proceeded in the inverse way. From Einstein's equations for the gravitational field and taking into account the Cosmological constant, the differential equation that describes the trajectory of a planet around the Sun was obtained [1]. Now, from the perihelion of each of the planets of the Solar System, the value of the Cosmological Constant for each of them was theoretically estimated. The results obtained are in agreement with those obtained by other authors [11, 12].

## 6. Conclusions

We have obtained the exact value for the precession of the perihelion of the planets of the Solar System. The algebraic formulas were obtained to calculate the displacement of the perihelion of each one of the planets of the Solar System. Using the differential equation related to the contribution of the cosmological constant, we obtained predictions about its value. Our results are consistent with others already published in the literature. We hope that this work and the proposed methodology will be of interest to theoretical physicists, astronomers, and cosmologists.

## Figures and Tables

**Table 1:** Planets data.

Planet	*A* : Apelion (meters)	*P*:Perihelion (meters)	Sidereal Period	$Q=\frac{P}{A}$
Mercury	69817332000	46000870000	87.969089	0.658875
Venus	108939198000	107475372 000	224.69562	0.986563
Earth	152100915000	147094882000	365.256622	0.967087
Mars	249226166000	206662107000	686.99329	0.829215
Jupiter	816054481000	740505444000	4334.24677	0.907422
Saturn	1506619721000	1348156111000	10765.21936	0.894822
Uranus	3004984160000	2735977617000	30700.24558	0.91048
Neptune	4538617181000	4458057447000	60226.53638	0.98225
Pluto	7377158662000	4437141859000	90631.02406	0.60147

**Table 2:** Exact and approximate values for perihelion shifts.

Planet	Δ_*GTR*_ (Exact Value)	${\tilde{\Delta }}_{GTR}$	${\hat{\Delta }}_{GTR}$	$\left|\Delta_{GTR}-{\tilde{\Delta }}_{GTR}\right|$	$\left|\Delta_{GTR}-{\tilde{\Delta }}_{GTR}\right|$
Mercury	42.981518799592365	42.981524756819105	42.98151983984209	5.96 × 10^−6^	1.04 × 10^−6^
Venus	8.625078587720143	8.625079290772037	8.625078762663101	7.06 × 10^−7^	1.75 × 10^−7^
Earth	3.8387718031722122	3.8387720280528486	3.8387718587113353	2.25 × 10^−7^	5.55 × 10^−8^
Mars	1.3508767421610324	1.3508767918831506	1.3508767534274582	4.97 × 10^−8^	1.13 × 10^−8^
Jupiter	0.062311625478272306	0.062311626166467286	0.06231162564333644	6.88 × 10^−10^	1.65 × 10^−10^
Saturn	0.013688932210705418	0.013688932292810001	0.013688932230249766	8.21 × 10^−11^	1.95 × 10^−11^
Uranus	0.0023848021538887348	0.0023848021610369903	0.002384802155606265	7.15 × 10^−12^	1.72 × 10^−12^
Neptune	0.000774085957956637	0.0007740859594722801	0.0007740859583331821	1.52 × 10^−12^	3.77 × 10^−13^
Pluto	0.0004175446900080054	0.00041754469056943497	0.0004175446900902417	5.61 × 10^−13^	8.22 × 10^−14^

**Table 3:** Prediction for a positive Einstein's cosmological constant.

Planet	Predicted Value Λ=Λ_+_
Mercury	1.02 × 10^−47^
Venus	6.34 × 10^−49^
Earth	1.05 × 10^−49^
Mars	5.82 × 10^−59^
Jupiter	3.0 × 10^−63^
Saturn	5.1 × 10^−55^
Uranus	2.79 × 10^−56^
Neptune	4.13 × 10^−57^
Pluto	1.1 × 10^−66^

## Data Availability

The data used may be found at https://calgary.rasc.ca/orbits.htm. This reference is cited within the manuscript.
